# Management of evidence and conflict of interest in guidelines on early childhood allergy prevention and child nutrition: study protocol of a systematic synthesis of guidelines and explorative network analysis

**DOI:** 10.12688/f1000research.123571.1

**Published:** 2022-11-10

**Authors:** Katharina Sieferle, Corinna Schaefer, Eva Maria Bitzer

**Affiliations:** 1 Department of Public Health and Health Education, Pädagogische Hochschule, Freiburg, 79117, Germany; 2 Agency for Quality in Medicine, Berlin, Germany

**Keywords:** Early childhood allergy prevention, clinical practice guideline, food-based dietary guideline, conflict of interest

## Abstract

**Background:** With the rising prevalence of allergic diseases in children, prevention of childhood allergies becomes an important public health issue. Recently, a paradigm shift is taking place in the approach to preventing allergies, and clinical practice guidelines (CPG) and food-based dietary guidelines (FBDG) play an important role in providing practitioners with the latest evidence and reliable guidance. However, concern about the methodological quality of the development of FBDGs and CPGs, including limitations in the systematic reviews, lack of transparency and unmanaged conflicts of interest (COI), reduce the trust in these guidelines.

**Methods:** We aim to synthesize the available guidance on early childhood allergy prevention (ECAP) through a systematic search for national and international CPGs and FBDGs concerning ECAP and child nutrition (CN) and to assess the quality of the guidelines and management of COI. Additionally, we will analyse the content and the evidence base of the recommendation statements. We aim to quantify the COI in guideline panellists and explore possible associations between COI and recommendations. Through a social network analysis, we expect to elucidate ties between panellists, researchers, institutions, industry and other sponsors. Guidelines are an important tool to inform healthcare practitioners with the newest evidence, but quality and reliability have to be high. This study will help identify potential for further improvement in the development of guidelines and the management of COI. If the social network analysis proves feasible and reveals more information on COI in comparison to disclosed COI from the previous analyses, the methodology can be developed further to identify undisclosed COIs in panellists.

**Ethics and dissemination:** This research does not require ethical approval because no human subjects are involved. Results will be published in international peer-reviewed open access journals and via presentations at scientific conferences.

## Introduction

The prevalence and impact of allergic diseases, including food allergies, eczema, contact dermatitis as well as hay fever and asthma is continuously rising, especially in children. ^1^ Asthma and other allergic diseases reduce the quality of life and their economic burden is high. ^1^^,^^2^ Therefore prevention of childhood allergies is an important public health concern. ^1^ Prevention of food allergies previously concentrated on avoidance of the potential allergen during pregnancy, breast-feeding and infancy, but based on the “dual-allergen-exposure hypothesis” ^3^ a gradual paradigm shift is taking place in the approach to preventing allergies - from avoidance to early and sustained exposure. According to this hypothesis, exposure to food allergens through the skin can lead to allergic sensitization whereas early consumption of the food protein can induce oral tolerance. ^3^ The hypothesis has been investigated in numerous trials with different objectives. Although the preventive effect of early introduction of peanuts and chicken eggs on food allergies has been confirmed in different systematic reviews, ^4^^,^^5^ there still remains uncertainty with respect to the definition of populations at risk and the preparation of food (e.g. boiled, pasteurised or raw egg). With regard to the prevention of eczema and asthma the findings are inconclusive ^6^^–^^8^ and additional studies are needed. Thus, despite extensive and ongoing research on early childhood allergy prevention (ECAP), the pieces of the puzzle revealed so far do not provide a comprehensive picture and several pieces are still missing.

In fields with rapidly evolving evidence such as ECAP, clinical practice guidelines (CPG) and food-based dietary guidelines (FBDG) play an important role in providing practitioners in allergy prevention and child nutrition (CN) with reliable guidance. FBDGs and CPGs are statements including recommendations intended to optimize health behaviour and patient care, which are ideally informed by systematic reviews of existing evidence and an assessment of the benefits and harm of alternative care options. ^9^^,^^10^ It should be noted that making guideline recommendations always involves judgement: regarding the strengths and limitations of the evidence, the balance of benefits and harm and ethical or legal considerations. ^11^ Thus, guidelines, though based on a systematic review, are far from “objective”. With respect to the prevention of food allergies the review of Perkin *et al*., ^12^ illustrates that guidelines interpret the evidence differently and come to diverging recommendation statements: While some institutions frame their statements according to the PICO-scheme of the underlying trials, i.e. the LEAP trial regarding the introduction of peanuts, ^13^ others assume the evidence can be extrapolated to a variety of food allergens beyond peanut and chicken eggs and advocate for their early introduction.

Even if our understanding of the impact of FBDGs and CPGs on preventive practice and public health outcomes is limited, they have the potential to enhance translation of research into practice, improve healthcare quality and safety ^9^^,^^14^ and shape the professional and public discussion on health and nutrition. ^15^

There is considerable concern from physicians, consumer groups and other stakeholders about the methodological quality of the development of FBDGs and CPGs, including limitations in the systematic reviews that serve as the evidence base for CPGs, lack of transparency and unmanaged conflicts of interest. ^16^^–^^20^ Diverging recommendations across guidelines might decrease the confidence in guidelines in general, if the reasoning leading to a recommendation statement is not transparent and no information regarding the developmental process of the guideline is provided. ^9^^,^^21^ Organizations such as the U.S. Institute of Medicine and the Guidelines International Network (G-I-N) have therefore developed recommendations to define trustworthy guidelines. ^14^

When assessed for their overall quality using the Appraisal of Guidelines for Research and Evaluation (AGREE II) tool, CPGs on for example the management of pediatric Type 2 Diabetes Mellitus and the management of fever in children only achieved insufficient scores in the domain ‘Rigor of development’, showing that the methodology of guideline development can still be improved. ^22^^,^^23^ However, to date there has been no systematic investigation of whether CPGs and FBDGs on ECAP comply with methodological standards in guideline development.

Conflicts of interest (COI) have also been identified as causing differences in recommendations ^16^^,^^19^^,^^24^ and in turn COI were suggested to be one of the most relevant factors impairing the public’s trust in nutrition guidelines. ^25^ In the context of guideline development COI can be understood as “circumstances that create a risk that professional judgements or actions regarding a primary interest will be unduly influenced by a secondary interest”. ^26^^,^^27^ Secondary interests can be divided into material interests, which include actions leading to direct financial gain, and non-material interests, which can include the pursuit of professional status and recognition, competition with other professionals, support of friends or colleagues, and access to or remaining in a group or network. COI can also be divided into direct financial COI, which arise from financial relationships with persons or organizations, including fees for lectures, investments or shares in products or services or study sponsorship, and into indirect COI. In addition to non-material COI, indirect COI also include intellectual COI, which refer to the increased risk of maintaining a specific point of view due to one’s own academic activities. ^28^

It has to be assumed that industry sponsorship is prevalent in ECAP ^29^^–^^31^ and with the paradigm shift in allergy prevention, commercial interest might even be increasing with regard to “baby food add-ins” which claim to contain adequate doses of allergens to facilitate introduction and induce oral tolerance. ^12^ Therefore, vested interests could be fostered as well. Standards for disclosure and management of COIs in guideline development have been set by the Guidelines International Network in 2015. ^28^ These recommendations include issues such as trying not to include members in the guideline development panel if they have (direct financial or indirect) COI, using standardized forms for the disclosure of interests, making disclosures of interests publicly available and easily accessible and others. ^28^ However, it has been shown that many guideline authors have potentially relevant undisclosed COI and COI management is often inadequate. ^28^^,^^32^^,^^33^ It is not yet known, to what extent policies and standards for management and disclosure of COI have been adopted for the development of guidelines on ECAP and whether this point is also acknowledged in FBDGs. ^33^^–^^35^ It is also not clear, whether COI in guideline development are associated with the content of the recommendations and with the prevalence of intervention- and industry-friendly recommendations.

### Objective

This study aims to synthesise national and international guidelines on ECAP and child nutrition and to systematically assess their methodological quality. We aim to investigate the management of conflicts of interest in these guidelines and the possible association of the COI with the guideline recommendations. We will also use the data to explore and visualize the networks of guideline panellists in the field of ECAP and child nutrition.

## Methods

The methods for the search strategy, selection and data collection processes will be reported based on the Preferred Reporting Items for Systematic Reviews and Meta-Analyses (PRISMA) guidelines on the conduct of systematic reviews. ^36^ This protocol is reported in line with the PRISMA-P guidelines. ^48^

The study consists of three consecutive tasks:

1. Assess the quality of the guideline development process and the management of evidence and COI of CPGs and FBDGs on ECAP and CN and provide a content analysis of the recommendation statements and an analysis of the evidence base of the recommendation statements.
2. Quantify the amount of COI in guideline panel members and explore the association between the role of the panellist and COI and between COI and the recommendation statements.
3. Explore the merits of social network analysis as a tool to elucidate ties between guideline panel members, ECAP and CN researchers, and research sponsors.

### Task 1

*Search strategy*

**Figure 1.**
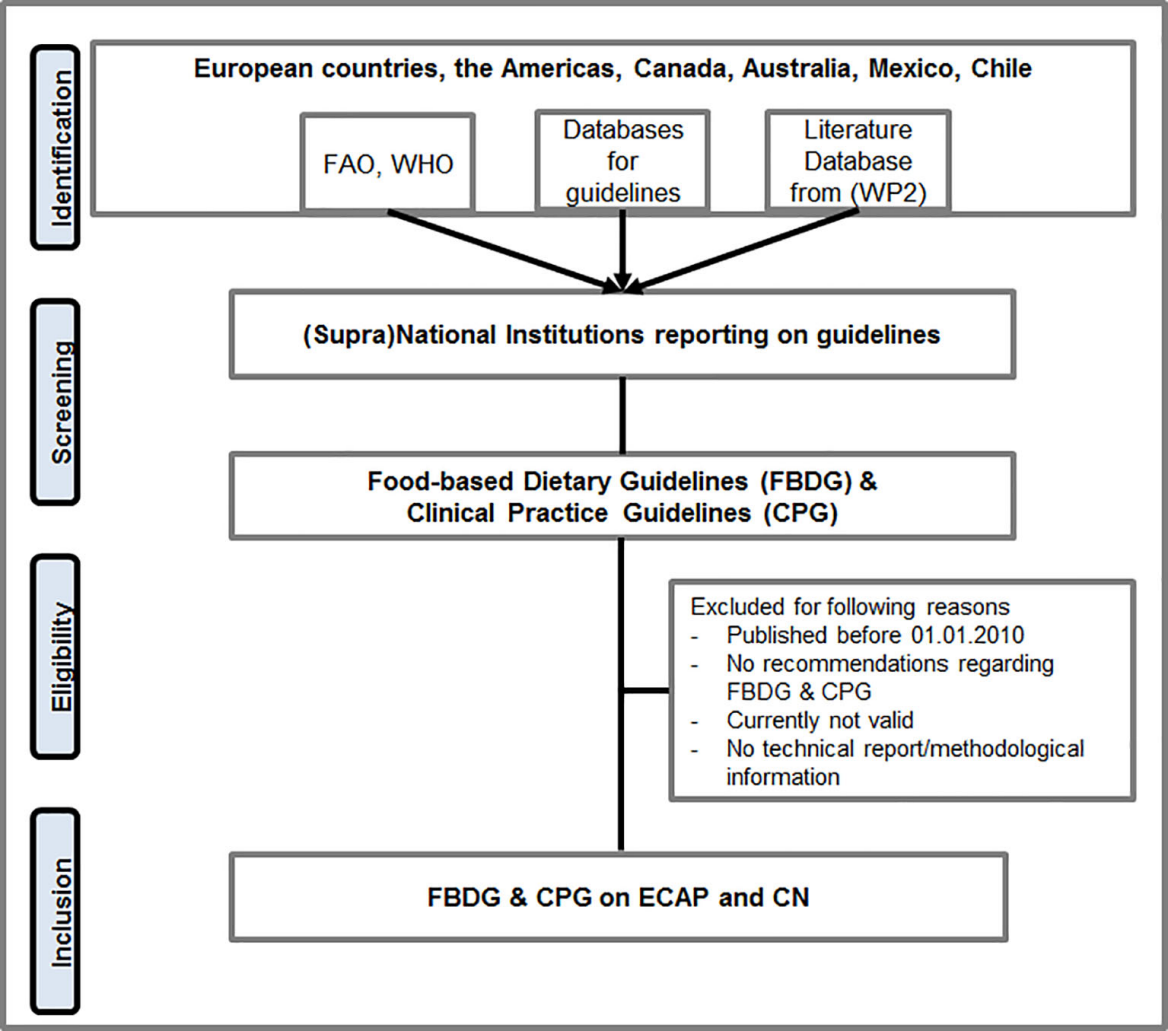
Search strategy. Legend: FAO=Food and Agriculture Organization of the United Nations, WHO=World Health Organization, WP2=Work package 2, FBDG=Food-based Dietary Guideline, CPG=Clinical Practice Guideline, ECAP=Early Childhood Allergy Prevention, CN=Child Nutrition.

Only a small percentage of guidelines is published in databases like MEDLINE, Embase etc. Therefore we will conduct the systematic search for national and international CPGs and FBDGs concerning ECAP and CN according to established recommendations for guideline retrieval ^37^ including the following databases and websites: Guideline International Networks database on clinical guidelines (GIN), Turning Research into Practice (TRIP), ECRI Guidelines Trust and the Alliance for the implementation of Clinical Practice Guidelines (AiCPG) – the successor of the National Guideline Clearinghouse, the Association of the Scientific Medical Societies in Germany (AWMF), WHO, the National Institute for Health and Care Excellence (NICE), as well as national and international clinical specialty societies. MEDLINE will be searched using the CADTH filter for guidelines. ^38^ Furthermore, we aim to identify (supra) national institutions that report on dietary and/or clinical guidelines. For institutions reporting on FBDGs we will use the Food and Agriculture Organization of the United Nations (FAO) directory of FBDGs, that provides links to more than 100 guidelines from all over the world, summary information in English as well as additional resources. ^39^ Figure 1 describes the search strategy.

These websites and databases do not have the same extensive search possibilities as bibliographic databases (like MEDLINE), but the following search filters will be applied if available: only fully published guidelines (no guidelines in development, withdrawn, etc.), publication year from 2010, publication in English or German. The search will be restricted to publications dating from 2010 up to now in order to capture the earliest guidelines that could have addressed the ongoing evidence shift as well as all recent guidelines.

*Eligibility criteria*

Screening of the retrieved publications will be done by two members of the study group. Disagreements will be resolved by discussion. Publications will be eligible for inclusion if they meet the following criteria:

• Population: Infants (up to one year) or pregnant women or breastfeeding women (with or without increased risk for the development of allergies or asthma)
• Intervention: Primary prevention of immediate or IgE-mediated allergies, atopic eczema or asthma
• Comparator: not applicable
• Outcome: New cases of immediate or IgE-mediated allergies, atopic eczema or asthma
• Time frame: dating from 2010, only guidelines that were valid at the time of search and the most recent updates
• Publication types: We will include full guidelines and possible addenda, consensus statements, as well as position papers which address health professionals
• Language: English or German

*Data extraction and quality assessment*

Data of included guidelines will be extracted by the first author and cross-checked by another author. Data will be entered into a relational database using MS Access which facilitates data export for quantitative analysis. The following data will be extracted from each guideline: Guideline editor, leading scientific societies, sponsorship, contact and person responsible, composition of guideline panel (name, academic title, institution, area of expertise or profession, their tasks in relation to the preparation of the guideline), independence of the coordinators and lead authors, declaration and assessment of COI, report of any potential sources of COI (explanation of potential conflicts of interest, assessment of conflicts of interest, and managing conflicts of interest), direct COI (e.g. financial, personal, institutional benefits), indirect COI (e.g. clinical, academic, personal interest, membership or function in expert association, clinical activities, publications, author or co-authorship, research projects, conducting clinical trials, leading participation in educational institutes, personal relationships with a representative of a company in the healthcare industry), imposed abstentions because of COI and external review of the guideline draft and for each relevant recommendation statement the topic of recommendation, recommendation statement in plain text, level of evidence and grade of recommendation will be assessed.

Furthermore, we will extract data regarding the evidence base of the relevant recommendation statements including information on authors, publication type (in case of primary studies: study type, risk of bias), country and sponsorship for each cited study.

Besides the management and disclosure of COI, a balanced and varied composition of the guideline panel and of stakeholders involved in the guideline development is important to prevent single persons or professions from having a disproportionate influence on the recommendations. We will therefore also assess the composition of the guideline panels and stakeholders involved in the development of the included guidelines.

To determine the methodological stringency of guideline development, AGREE II will be employed. The methodological quality of each included guideline will be independently assessed by two authors. AGREE II consists of 23 items covering the following 6 domains: scope and purpose, stakeholder involvement, stringency of development, clarity of presentation, applicability and editorial independence. Additionally, 2 overall assessment items are included: The quality of the overall guideline rated on a scale from 1-7 and the decision as to whether the guideline would be recommended for use in practice. The AGREE II overall assessment of the guideline indicates the general quality of the guideline. It “requires the user to make a judgement as to the quality of the guideline, taking into account the criteria considered in the assessment process” [40 S.10]. Each item of the AGREE II instrument is scored on a 7-point scale (1=strongly disagree to 7=strongly agree). Quality scores of each domain will be calculated, by adding up the scores of the individual items in the domain and by scaling the obtained score as a percentage of the maximum possible score for that domain:

$$\frac{\text{obtained score}-\text{minimum possible score}}{\text{maximum possible score}-\text{minimum possible score}}$$

The six domain-scores are independent and shall not be aggregated into a single quality score. ^40^ Discrepancies in scorings will be resolved by discussion between the two authors (KS, EMB).

*Synthesis of results and analysis*

The basic information of all included guidelines will be summarized narratively. Subsequently, guidelines will be categorized according to indications (e.g. food allergies, asthma, atopic eczema) to allow for content-related analysis within each group. Particular emphasis will be put on the respective comparisons of clinical practice guidelines regarding the prevention of food allergies in contrast to FBDG. First, we will compare the evidence base of recommendations. To this end, included publication types and number of citations will be analysed. In addition, the publications will be compared to the studies that have been included in a living systematic review of a neighbouring project within the HELICAP research group. ^41^ To complete the synthesis of guidelines, we will compare the recommendations issued by the different guidelines regarding the topic and tenor of each statement as well as the assigned level of evidence, and the respective grade of recommendation.

### Task 2

The second part of the analysis will be dedicated to the disclosure and management of COI. We will investigate what kind of and how often particular COI are disclosed, what measures are taken to manage COI and we want to explore whether associations exist between topics and COI. If possible, the analysis will be carried out on the level of recommendation statements, otherwise information will be summarized per guideline. Then this analysis will be repeated, this time using the guideline authors as a unit of analysis. In order to capture relevant research activities of guideline panellists a co-author analysis will also be carried out: Authors and co-authors of studies that have been included in the living systematic review of our neighbouring project within HELICAP will be matched to a list of guideline panellists. ^41^

### Task 3

In the third part of the analysis we will visualize collaboration networks between guideline panellists, researchers and research sponsors using a social network analysis. ^42^ Authors, institutions, and sponsors will be understood as “actors” and connections between them such as affiliations or joint publications will inform the “ties” between them. ^42^ Different metrics of density and centrality indices will be employed to represent relationships and patterns of interaction. ^43^ The strength of ties between two actors will be determined according to different indicators, e.g. the number of joint publications of two researchers or the funding a scientist received from a particular company within a particular time period. ^43^ These indicators are designed to identify core-actors (opinion-leaders), subgroups with closer relationships (cluster or cliques) as well as actors who serve as “bridges” and connect otherwise separated actors and clusters within a network. ^44^^–^^46^ Moreover, in a small pilot study, e.g. for German guideline authors, we will investigate the impact of additional information, e.g. congress programs or entries in study registries, on the shape of the networks.

For content-related analysis MAXQDA 2020, a software used in qualitative studies, will be employed. All statistical analysis will be conducted using SPSS Statistics 27. The network analysis will be carried out with Ucinet 6. ^47^

*Patient and public involvement*

Patients and/or the public were not involved in the design, conduct or reporting of this research.

## Discussion

Allergy prevention in early childhood is a topic that warrants collaborative public health action as it affects the daily routine of parents in the areas of nutrition and environmental exposure. It involves different areas of medical expertise, and it requires policies for example regarding the reduction of air pollution and the development of a healthy environment. To our knowledge, this will be the first study synthesizing the internationally available guidance on the primary prevention of IgE-mediated allergies, atopic eczema and asthma in early childhood. The findings will facilitate an integrated perspective on this topic and can help promote a collaborative discussion.

There is evidence that often COI are not disclosed adequately, ^32^ therefore we want to assess the number of COI disclosed in guidelines on ECAP. Assuming, that guideline authors disclose possible COI and that the respective guidelines report these disclosures adequately, we will also be able to provide a comprehensive overview of existing COI in the field of ECAP. In addition, we will determine whether there are associations between the COI and the content of the guideline recommendations. To do this, we will evaluate the COI of guideline panel members in guidelines with conflicting recommendations.

On the one hand potential for further improvement in the management of COI in guidelines will be identified and on the other hand transparency and the reliability of the guidelines can be increased. Furthermore, we expect a new understanding of research networks in ECAP and possible sources of COI as relationships between the actors of a network become evident. For example, if a researcher receives regular funding from one sponsor, we have to assume a high risk of a financial COI or if two researchers seem to collaborate closely based on joint publications, we have to assume a high risk of indirect COI. If the social network analysis proves feasible and reveals more information on COI in comparison to disclosed COI from the previous analyses, the methodology will be developed further.

### Ethics and dissemination

According to the ethic committee of the Chamber of Physicians Baden-Württemberg this research does not require an ethical approval because no human subjects are involved. Findings from this study will be published upon finalization in internationally peer-reviewed journals with focus on public health, allergy prevention and evidence-based medicine. The project consortium also establishes a plan to coordinate the participation of subprojects in national and international conferences of medical associations, e.g. European Academy of Allergy and Clinical Immunology, and German Society for Social Medicine and Prevention. When we publish our results, we do not mention any real names, so no conclusions can be drawn about individual persons.

### Study status

At submission of this protocol the systematic search and screening of search results has been completed. The quality assessment of the guidelines through AGREE II has been finalized and the data extraction on management and disclosure of conflicts of interests is underway.

## Data Availability

No underlying data are associated with this article. Figshare: PRISMA-P checklist for “Management of evidence and conflict of interest in guidelines on early childhood allergy prevention and child nutrition: study protocol of a systematic synthesis of guidelines and explorative network analysis”. https://doi.org/10.6084/m9.figshare.21501462.v1. ^48^ Data are available under the terms of the Creative Commons Attribution 4.0 International license (CC-BY 4.0).
