# Supplementary material for: Management of evidence and conflict of interest in guidelines on early childhood allergy prevention and child nutrition: study protocol of a systematic synthesis of guidelines and explorative network analysis
Source: F1000Res. 2023 Dec 27;11:1290. Originally published 2022 Nov 10. [Version 2] doi: 10.12688/f1000research.123571.2 (PMC10794862; doi:10.12688/f1000research.123571.2)
Supplement: Peer review history

# Reviewer report: Reviewer response for version 2

**DOI:** 10.5256/f1000research.160241.r233757

**Published:** 2024-01-17

**Authors:** Paxton Loke

**Affiliations:** 1 Allergy Immunology, Murdoch Children’s Research Institute, Parkville, Victoria, Australia

No further comments.

Is the study design appropriate for the research question?

Yes

Is the rationale for, and objectives of, the study clearly described?

Yes

Are sufficient details of the methods provided to allow replication by others?

Yes

Are the datasets clearly presented in a useable and accessible format?

Not applicable

Reviewer Expertise:

Food allergy

I confirm that I have read this submission and believe that I have an appropriate level of expertise to confirm that it is of an acceptable scientific standard.

# Reviewer report: Reviewer response for version 2

**DOI:** 10.5256/f1000research.160241.r233756

**Published:** 2024-01-11

**Authors:** Nina Veetnisha Gunnarsson

**Affiliations:** 1 School of Health and Welfare, Jönköping University, Jönköping, Sweden

I am happy with the changes made by the authors and I do belive that reducing the amount of documents will make this review stronger. This review will make a strong constribution to the topic.

Is the study design appropriate for the research question?

Yes

Is the rationale for, and objectives of, the study clearly described?

Yes

Are sufficient details of the methods provided to allow replication by others?

Partly

Are the datasets clearly presented in a useable and accessible format?

Yes

Reviewer Expertise:

NA

I confirm that I have read this submission and believe that I have an appropriate level of expertise to confirm that it is of an acceptable scientific standard.

# Reviewer report: Reviewer response for version 1

**DOI:** 10.5256/f1000research.135689.r206646

**Published:** 2023-09-15

**Authors:** Nina Veetnisha Gunnarsson

**Affiliations:** 1 School of Health and Welfare, Jönköping University, Jönköping, Sweden

This is a very innovative and interesting systematic review and synthesis of child allergy guidelines.

1. What is meant with immediate allergies? Clarify.
2. In figure 1, why were these countries included specifically and not others?
3. Population: infants up to one year? Rational behind this?
4. A replication of the study may be difficult, as there are many different analysis and tasks.
5. Time frame: from 2010, rational behind this choice?
6. Publication types: additional type of texts such as position papers, consensus statements etc, is this needed, and if, why?  

Is the study design appropriate for the research question?

Yes

Is the rationale for, and objectives of, the study clearly described?

Yes

Are sufficient details of the methods provided to allow replication by others?

Partly

Are the datasets clearly presented in a useable and accessible format?

Yes

Reviewer Expertise:

Allergy, food allergy

I confirm that I have read this submission and believe that I have an appropriate level of expertise to confirm that it is of an acceptable scientific standard, however I have significant reservations, as outlined above.

# Response

**Published:** 2023-12-20

**Authors:** Katharina Sieferle

**Affiliations:** Pädagogische Hochschule Freiburg, Germany

1. What is meant with immediate allergies? Clarify.

*We have originally used the phrase immediate allergies and IgE-mediated allergies in the study protocol, both meaning type I hypersensitivity reactions. We have decided to now only use IgE-mediated allergies to prevent any possible confusion or misunderstanding.*

 2. In figure 1, why were these countries included specifically and not others?

*This was originally decided due to feasibility reasons, expecting most English-speaking guidelines to originate from the included countries. After additional consideration, we have decided not to exclude other countries, and to instead include all available guidelines in English.*

*The search strategy has been updated and a new figure 1 has been included.*

 3. Population: infants up to one year? Rational behind this?

*The topic of interest of the research group is allergy prevention in early childhood and infancy. The first year of life is an important time for allergy prevention, since many allergy prevention measures concern the duration of breastfeeding and (timing of) introduction of complementary feeding, which typically is recommended to take place in the first year of life.*

*To be included in our analysis, guidelines do not need to exclusively address children under 1 year of life but should make recommendations on the first year of life, or on interventions in the mother during pregnancy.*

*The text was changed in the manuscript to make this clearer.*

 4. A replication of the study may be difficult, as there are many different analysis and tasks.

*This study is part of a German research group and is divided into three tasks. This study protocol covers the whole study with all included tasks. However, all tasks will be conducted and analyzed individually, making replication of the tasks easier.*

 5. Time frame: from 2010, rational behind this choice?

*Even though as early as 2008 avoidance of common allergens to prevent allergies was no longer recommended, there was a lack of evidence on allergy prevention measures (Greer et al. 2008). The LEAP study was published in 2015, leading to a further evidence shift towards an early introduction of common allergens (Du Toit et al. 2015). With guidelines from 2010 to 2019, we aim to be able to see this resulting shift in recommendations and underlying evidence. Additionally, only valid guidelines should be included in the analysis. Many guideline-developing organizations have set timeframes for guidelines to be valid for after publication. Guidelines older than 2010 should therefore definitely not be valid anymore at the time of search.*

*We have included a more comprehensive rationale in the text.*

 6. Publication types: additional type of texts such as position papers, consensus statements etc, is this needed, and if, why?  

*Thank you for this question/suggestion.*

*After additional consideration, we have decided to only include Clinical Practice Guidelines and Food-based Dietary Guidelines that make recommendations on Early Childhood Allergy Prevention, to compare these two publication types.*

*Eligibility criteria were changed accordingly in the manuscript.*

# Reviewer report: Reviewer response for version 1

**DOI:** 10.5256/f1000research.135689.r202322

**Published:** 2023-09-12

**Authors:** Paxton Loke

**Affiliations:** 1 Allergy Immunology, Murdoch Children’s Research Institute, Parkville, Victoria, Australia

This is an interesting study proposal in particular the conflict of interest aspect. Below are a few comments for clarification.

1. The search strategy in Figure 1 did not include Asia or Asian countries. Please comment on why these countries are excluded.
2. Please comment on including German publications and not any other languages besides English.
3. Population: Please comment on the rationale of including only infants up to one year and whether or not this may be overly restrictive.
4. Will the definition of IgE-mediated allergies, atopic eczema or asthma be included in the final publication?
5. Regarding Task 3: Please comment on whether or not there will be any bias and how this will be addressed. For example, it is possible that authors may collaborate to harness different expertise for a common goal, and this may not necessary be a conflict of interest.
6. Minor comment in Introduction: suggest deleting “only” from this sentence “…children only achieved insufficient scores…”

Is the study design appropriate for the research question?

Yes

Is the rationale for, and objectives of, the study clearly described?

Yes

Are sufficient details of the methods provided to allow replication by others?

Yes

Are the datasets clearly presented in a useable and accessible format?

Not applicable

Reviewer Expertise:

Food allergy

I confirm that I have read this submission and believe that I have an appropriate level of expertise to confirm that it is of an acceptable scientific standard, however I have significant reservations, as outlined above.

# Response

**Published:** 2023-12-20

**Authors:** Katharina Sieferle

**Affiliations:** Pädagogische Hochschule Freiburg, Germany

The search strategy in Figure 1 did not include Asia or Asian countries. Please comment on why these countries are excluded.

*-> This was originally decided due to feasibility reasons, expecting most English-speaking guidelines to originate from the included countries. After additional consideration, we have decided not to exclude Asian countries or other countries, and to instead include all available guidelines in English.*

*The search strategy has been updated and a new figure 1 has been included.*

 Please comment on including German publications and not any other languages besides English.

*-> We included German guidelines because this study is part of a German research group on Public Health, making the German perspective especially relevant.*

*Besides German, only guidelines in English were included, due to feasibility and limited resources.*

 Population: Please comment on the rationale of including only infants up to one year and whether or not this may be overly restrictive.

*-> The topic of interest of the research group is allergy prevention in early childhood and infancy. The first year of life is an important time for allergy prevention, since many allergy prevention measures concern the duration of breastfeeding and (timing of) introduction of complementary feeding, which typically is recommended to take place in the first year of life.*

*To be included in our analysis, guidelines do not need to exclusively address children under 1 year of life but should make recommendations on the first year of life, or on interventions in the mother during pregnancy and breastfeeding. We therefore think this will not be too restrictive.*

*The text was revised in the manuscript to make this clearer.*

 Will the definition of IgE-mediated allergies, atopic eczema or asthma be included in the final publication?

*-> So far, we had not included a definition, but upon your question have now included further references, on which we have based our understanding of allergic diseases, in the study protocol.*

 Regarding Task 3: Please comment on whether or not there will be any bias and how this will be addressed. For example, it is possible that authors may collaborate to harness different expertise for a common goal, and this may not necessary be a conflict of interest.

*-> We are not sure if we understood this comment correctly.*

*Ideally, a guideline panel should encourage authors from different areas of expertise that are relevant to the subject area of the guideline, as well as patients or citizens to collaborate. A diverse guideline panel facilitates the consideration of different perspectives and helps avoid possible distortions caused by secondary interests.*

*Conflicts that might arise from different expertise belong to the category of intellectual conflicts of interest, which we don’t see as severe. We are primarily interested in direct financial and personal interests, to what extent they occur and if they occur, how they are managed.*

*We hope this answered your question?*

 Minor comment in Introduction: suggest deleting “only” from this sentence “…children only achieved insufficient scores…”

*-> Thank you for the suggestion, the text was changed accordingly.*
